# Pd Doped Co_3_O_4_ Loaded on Carbon Nanofibers as Highly Efficient Free-Standing Electrocatalyst for Oxygen Reduction and Oxygen Evolution Reactions

**DOI:** 10.3389/fchem.2021.812375

**Published:** 2022-01-12

**Authors:** Ruyue Wang, Deshuang Hu, Peng Du, Xiaodi Weng, Haolin Tang, Ruiming Zhang, Wei Song, Sen Lin, Kai Huang, Ru Zhang, Yonggang Wang, Dongyu Fan, Xuchao Pan, Ming Lei

**Affiliations:** ^1^ State Key Laboratory of Information Photonics and Optical Communications and School of Science, Beijing University of Posts and Telecommunications, Beijing, China; ^2^ Beijing Key Laboratory of Space-ground Interconnection and Convergence, Beijing University of Posts and Telecommunications (BUPT), Beijing, China; ^3^ Unit 96911 of PLA, Beijing, China; ^4^ Guangdong Hydrogen Energy Institute of WHUT, Foshan, China; ^5^ Foshan Xianhu Laboratory of the Advanced Energy Science and Technology Guangdong Laboratory, Foshan, China; ^6^ School of Physical Science and Technology, Guangxi University, Nanning, China; ^7^ Ministerial Key Laboratory of ZNDY, Nanjing University of Science andTechnology, Nanjing, China

**Keywords:** electrospinning, doping, nanofiber, self-supporting electrode, bifunctional

## Abstract

Self-supporting electrodes usually show excellent electrocatalytic performance which does not require coating steps, additional polymer binders, and conductive additives. Rapid *in situ* growth of highly active ingredient on self-supporting electric conductors is identified as a straight forward path to prepare binder-free and integrated electrodes. Here, Pd-doped Co_3_O_4_ loaded on carbon nanofiber materials through electrospinning and heat treatment was efficiently synthesized, and used as a free-standing electrode. Benefiting from its abundant active sites, high surface area and effective ionic conduction capability from three-dimensional (3D) nanofiber framework, Pd-Co_3_O_4_@CNF works as bifunctional oxygen electrode and exhibits superior activity and stability superior to commercial catalysts.

## Introduction

To date, a wide range of electrochemistry technologies have received extensive attention in both academia and industry. In the extensive literature, most reported catalysts for electrochemistry are synthesized in powder form, and then coated on the current collector (electrode substrate) by adding a polymer binder (such as Nafion or polytetrafluoroethylene) (Huang et al., 2021; Yang et al., 2021a). Unfortunately, catalysts especially with nanostructure are easy to disperse unevenly and aggregate during casting to deteriorate electrocatalytic activity (Li et al., 2019a; Zhao et al., 2020). Moreover, the active materials are usually peeling-off from the electrode substrate during the reaction process of gas evolution or reduction as a result of the weak bonding between active components and carrier (Li et al., 2018). Furthermore, the non-conductive binder inevitably covers the active sites, limits the electrons conductivity, and increases the inner resistances of electrodes, which will determine the performance of battery by influencing the charge and mass transfer rate of the electrode (Chen et al., 2019; Jiang et al., 2020).

In contrast, recent studies demonstrate that self-supporting electrodes show excellent electrocatalytic performance which do not require coating steps, additional polymer binders, and conductive additives (Tong et al., 2018; Zhang et al., 2018; Huang et al., 2020a). Flexible carriers such as metal foam or other carbon based fluid collection are commonly used to obtain electrodes with mechanical strength (Nie et al., 2018; Li et al., 2021; Yang et al., 2021b). The introduction of additional substrates can undoubtedly increase the mechanical strength, but the compact structure of substrates inevitably leads to poor air/electrolyte permeability, thereby reducing the performance of electrodes (Du et al., 2021; Song et al., 2021). Therefore, it is difficult but of great significance to manufacture integrated electrodes with high activity. Rapid *in situ* synthesis of highly active ingredient on self-supporting electric conductors is identified as a straight forward path to prepare such binder-free and integrated electrodes. Thanks to its high surface area, outstanding flexibility and electrical properties, one-dimensional (1D) structure of carbon nanofibers have been extensively applied for energy storage and conversion equipment as high efficiency electrodes (Li et al., 2017; Kuang et al., 2018; Ran et al., 2018). More importantly, nanofibers could be easily assembled into three-dimensional (3D) conductive nanofiber framework which exhibits unparalleled advantages (Xiao et al., 2021). When used as an independent electrode in energy device, it will facilitate contact and conduction between the active components and ions, benefitting from a lot of pores and tracks in the framework that can store the electrolyte.

Besides, the intrinsic electrocatalyst activity is also significant to manufacture high performance electrodes (Li et al., 2020; Xiao et al., 2021). Cobalt oxide has been studied extensively, because of their sustainability against corrosion, outstanding redox capability, distinct 3 days electron orbitals, and superior theoretical activity (Xiang et al., 2018; Yu et al., 2018; Huang et al., 2020b; Hou et al., 2020; Gao et al., 2021; Liu et al., 2021). Although there are many studies on cobalt oxide nanofibers (Huang et al., 2019a; Li et al., 2019b), it is still facing great challenges to directly use it as a self-supporting electrode because of its lack of mechanical strength. But compounding with carbon materials will be a feasible way to make up for its deficiency. By combining it with nitrogen-doped graphite carbon fixed in the nanofiber, Huo et al. anchored cobalt nanoparticles in the carbon fiber and proved that it could be used as self-supporting air electrode in Zn-air battery (Chen et al., 2020). However, the activity of cobalt oxide and carbon composite electrodes is still too poor for energy equipment applications and has remarkable room for improvement. Anyway, noble metal modification is regarded as an effective method to improve the electrocatalytic performance by adjusting the composition and electronic properties of catalysts (Huang et al., 2019b). Due to the close lattice structure, Pd is considered to have comparable performance with Pt in electrochemical applications (Cheng et al., 2020). Riley synthesized dodecagon N-doped PdCoNi carbon-based nanosheets demonstrating superior performance for electrocatalytic water splitting and suitable for wide pH electrolytes (Wang et al., 2021). Thus, combining Pd with composite substrates could significantly improve the proton transfer rate, reaction kinetics, thereby effectively enhancing the catalytic performance.

In our work, we designed a Pd-doped Co_3_O_4_ loaded carbon nanofiber (Pd-Co_3_O_4_@CNF) electrode. Considering the simplicity and efficiency of electrospinning to prepare nanofibers, combined with the heat treatment process, we have achieved the preparation of the Pd- Co_3_O_4_@CNF electrode. Such electrode shows extraordinary oxygen reduction reaction (ORR) and oxygen evolution reaction (OER) performance in alkaline solution, and exhibits superior zinc-air battery (ZAB) performance to commercial catalysts.

## Experimental Section

### Material Synthesis

Pd-Co_3_O_4_@CNF was prepared by electrospinning and subsequent thermal treatment (Li et al., 2015; Zhang et al., 2021). The precursor solutions for electrospinning were prepared by dissolving 1 g PAN (Mw = 150,000), 0.016 g Palladium (II) acetylacetonate (C_10_H_14_O_4_Pd) and 0.23 g cobalt acetate (Co(AC)_2_·4H_2_O) in 10 g dimethylformamide (DMF) under magnetically stirring for over 5 h. The obtained precursor was filled in a syringe with 23-gauge blunt tip needle. Electrospinning was carried out under the electrostatic filed of 22 kV (Xie et al., 2020). The rotating speed of the collector is 200 RPM, and the distance between the nozzle and collecting carrier is 26 cm. The outflow rate of syringe pump is 0.1 ml min^−1^. After 8 h, the composite film was collected.

For carbonization, under argon flow, the composite film was first stabilized 250°C for 1 h, then increased to 900°C at 5°C min^−1^ and stayed for 1 h, and then obtained black film. Subsequently, the black film reheated to 200°C in muffle under air for 1 h to get Co_3_O_4_ phase. Finally, Pd-Co_3_O_4_@CNF was obtained.

### Characterization

The crystal structure of samples was investigated by an X-ray diffractometer (XRD, D/max 2500 V). A scanning electron microscope (SEM, Zeiss Ultra Plus), transmission electron microscopy (TEM), and high-resolution transmission electron microscopy (HR-TEM) were used to analyze the microstructure. Distribution of elements in pd-Co_3_O_4_@CNF was obtained by the energy dispersive spectrometer (EDS). We used X-ray photoelectron spectroscopy (XPS, Escalab 250 Xi) to study the surface chemistry of these samples.

### Electrochemical Characterization

All the electro-catalytic tests were tested using electrochemical workstations (Shanghai Chenhua Instrument Co. Ltd.CHI660E and CHI760E). The electrolyte is 1 M KOH solution. The counter electrode and reference electrode are graphite rod and Hg/HgO (filling with 1M KOH) respectively. All potentials are calibrated to reversible hydrogen electrodes (RHE) on base of the next equation: *E*_*RHE*_ = *E*_*Hg/HgO*_ + 0.098 + 0.059×pH. All powder catalysts for comparison will be prepared as slurry coated on the substrate as a working electrode. To test ORR performance, the prepared electrode film was cut into a circle with diameter of 5 mm and fixed on the surface of the rotating disk electrode by Nafion solution. To study the OER performance, the catalyst film was cut into rectangular pieces of 1.5 × 0.5 cm^2^, and used directly as a working electrode.

For ORR, 30 cycles of cyclic voltammetry (CV) tests were performed at a scanning rate of 50 mV s^−1^ in N_2_ saturated electrolyte for activation. Linear sweep voltammetry (LSV) test was then performed at 1600 RPM in an O_2_ saturated electrolyte with scanning rate of 10 mV s^−1^. The accelerated durability test (ADT) was carried out at voltage from 0.57 to 1.07 V (*vs.* RHE) for 5000 cyclic voltammetry cycles with sweep rate of 100 mV s^−1^. Nyquist plots were tested by electrochemical impedance spectroscopy (EIS) measurements in O_2_-saturated electrolyte at 0.85 V (*vs*. RHE). For OER, 30 cycles CV tests were performed at a scanning rate of 50 mV s^−1^ in N_2_ saturated electrolyte for activation. LSV test was then performed in N_2_ saturated electrolyte with sweep rate of 10 mV s^−1^. The ADT were carried out at the voltage range of 1.02–1.52 V (*vs.* RHE) for 5000 cyclic voltammetry cycles with a scan rate of 100 mV s^−1^. Nyquist plots was tested at 1.6 V (*vs*. RHE) in N_2_-saturated electrolyte. The stability tests were further investigated by long-term chronoamperometry (CA) tests for 20 h.

### Assembly and Test of Zn-Air Batteries

Zn foil was used as the anode. Air cathode is assembled by an air diffusion layer, foamed nickel, and a catalyst layer. Obtained Pd-Co_3_O_4_@CNF sample was cut in a 1.5 × 2 cm^2^ rectangular piece (5 mg in total, Pd mass loading of 0.25 mg) to make a catalyst layer. For the comparative commercial sample (Pd/C + RuO_2_), air electrodes loaded with equal mass of Pd were prepared by drip coating using the same ORR catalyst formulation ratio. The electrolyte was contained by 6 M KOH and 0.2 M ZnCl_2_ solutions. LANBTS BT-2016C system was used for cycling test (20 min for each charge and discharge period).

## Results and Discussion

**SCHEME 1:**
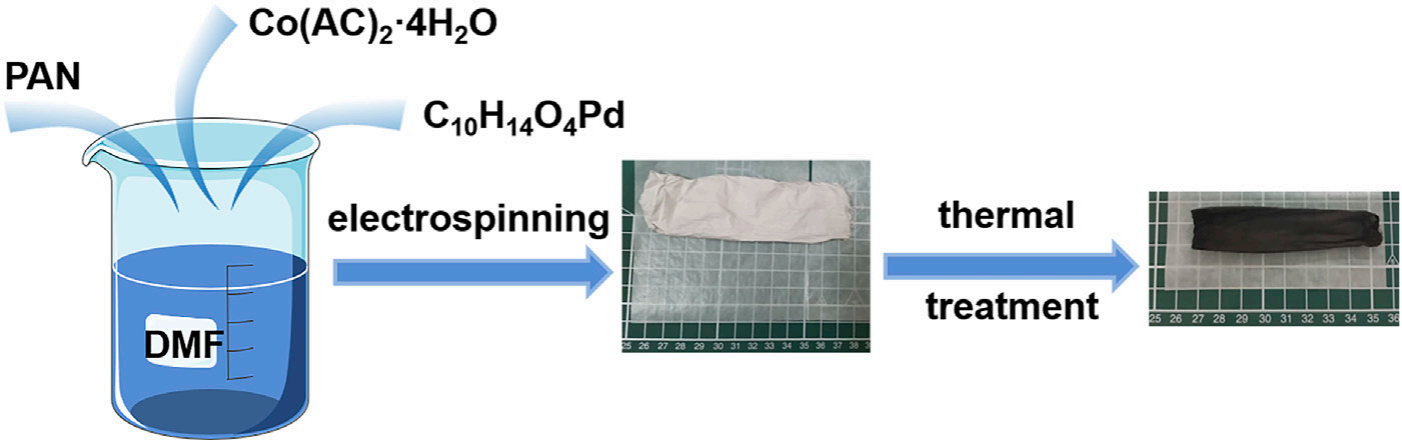
Schematic of the synthetic procedure of Pd-Co_3_O_4_@CNF.

**FIGURE 1:**
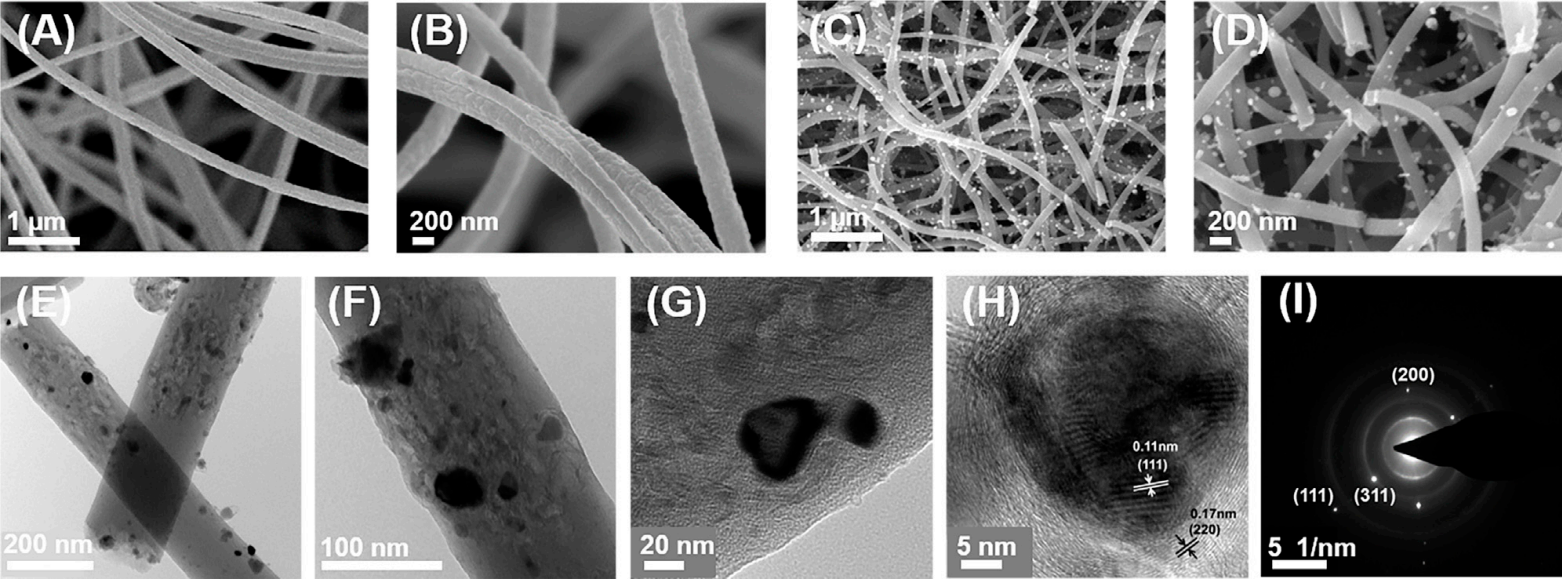
**(A,B)** SEM images of nanofibers before heat treatment. **(C,D)** SEM images of nanofibers after heat treatment. **(E–H)** TEM and **(I)** SAED images of Pd-Co_3_O_4_@CNF.

The fabrication process of Pd-Co_3_O_4_@CNF is illustrated in Scheme 1. Pd-Co_3_O_4_@CNF was successfully obtained *via* an ordinary electrospinning method and subsequent heat treatment. (Details are shown in the experimental section.) As a comparative sample, Co_3_O_4_@CNF was synthesized in the same way except that C_10_H_14_O_4_Pd was not added. In the experiment a flexible fiber membrane with a width of about 11 cm was obtained after electrospinning, and a 9.5 cm wide self-supporting electrode with mechanical strength still can be obtained by subsequent heat treatment. SEM and TEM characterization were performed to directly observe nanoscale structure of samples. As demonstrated in Figures 1A,B, the diameter of uniform nanofibers was about 200 nm and constituted the main mechanics of flexible fabric structure. Pd and Co_3_O_4_ are randomly incorporated in the fibrous structure. After heat treatment, nanoparticles can be observed uniformly distributed on the surface of the nanofiber (Figures 1C,D and Supplementary Figure S1). Moreover, some fibers are broken after heat treatment which may be due to the transition from Co phase to Co_3_O_4_ phase during the heat treatment process and part of the Co_3_O_4_ nanoparticles are formed. Direct visualization by TEM demonstrates that our approach successfully achieves the preparation of Pd-Co_3_O_4_@CNF composites. TEM images in Figures 1E–H exhibit a specific structure of nanofibers containing holes and nanoparticles. And the observed lattice distances of 0.11 and 0.17 nm (Figure 1H) can be indexed to (111) and (220) planes of Co_3_O_4_ crystal. This also proves the formation of cobalt oxide nanoparticles. As shown in Figure 1I **,** the fast Fourier transform (FFT) pattern was further displayed, corresponding to the crystal structure of Co_3_O_4_. (Li et al., 2019b). In addition, EDS element mapping of C, Co, O, and Pd elements also reveal the uniform distribution of Pd and Co_3_O_4_ (Supplementary Figure S2). Hence, the characterization results of the morphologic structure confirm that Pd-Co_3_O_4_@CNF conductive framework is successfully prepared by design as expected.

**FIGURE 2:**
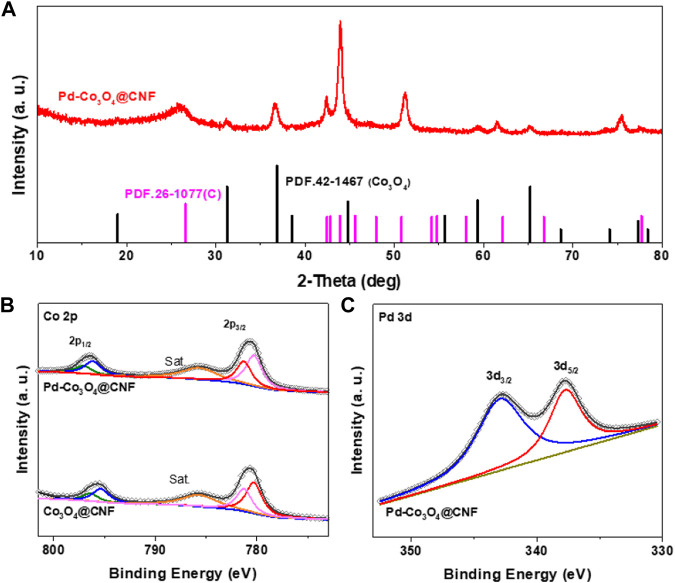
**(A)** XRD patterns of synthesized Pd-Co_3_O_4_@CNF. XPS spectra of **(B)** Co 2p and **(C)** Pd 3 days.

In order to clarify the crystal structure, chemical composition and element coordination of the electrodes, XRD, and XPS were further characterized. As shown in Figure 2A, all diffraction peaks of Pd-Co_3_O_4_@CNF can be accurately corresponded to the standard card (Co_3_O_4_, PDF.42-1467) and (C, PDF.26-1077), suggesting the high purity and crystalline. Moreover, in the XRD pattern, there are no diffraction peak which can be attributed to palladium or palladium oxides, suggesting that no large Pd-based nanocrystals exist in Pd-Co_3_O_4_@CNF sample and ultra-low doping of Pd will not change the phase of Co_3_O_4_. To further investigate the valence state of the Pd-Co_3_O_4_@CNF, together with the Co_3_O_4_@CNF, XPS measurements were performed. As shown in the Co 2p spectrum (Figure 2B **)**, the peaks are located at 780.0 and 795.5 eV, 781.4 and 797.2 eV, demonstrating the existence of Co^2+^ and Co^3+^ species, respectively (Lv et al., 2019; Huang et al., 2020c). Compared to Co_3_O_4_@CNF, the Co 2p_1/2_ of Pd-Co_3_O_4_@CNF moves towards a position with higher binding energy due to the doping of Pd influences the combination of Co and O, which will facilitate the migration of reactive oxygen species (Feng et al., 2021). As shown in Figure 2C, fitted Pd 3 days pattern of Pd-Co_3_O_4_@CNF exhibits peaks of 337.5 and 342.8 eV, which corresponded to the 3d_5/2_ and 3d_3/2_ spin orbit constituents of Pd specie (Du et al., 2020). This proves that Pd is successfully doped in the form of Pd^2+^, which will inevitably affect the combination of Co and O. As illustrated in C 1s pattern (Supplementary Figure S3), peaks correspond to the following groups: carbon sp^2^ (C=C, 284.8 eV), carbon sp^3^ (C-C, 285.4 eV), epoxy/hydroxyls (C-O, 286.3 eV), and carboxylates (O-C=O, 288.9 eV), respectively (Huang et al., 2019a). The deconvoluted O 1s spectra (Supplementary Figure S4) exhibit four peaks at 531.0, 532.0, 533.5, and 536.9 eV, ascribing to the following functional groups: lattice oxygen, oxygen bonds in C=O, C-O, and adsorbed water (Abouali et al., 2015). In conclusion, the XPS survey spectrum **(** Supplementary Figure S5 **)** of the Pd-Co_3_O_4_@CNF reveals peaks of Pd, Co, C, and O, which is consistent with the expected from the sample preparation step and results of EDS elemental mapping. Moreover, as shown in Supplementary Table S1, the surface weight fraction of Pd from XPS is 1.94% and the bulk weight fraction of Pd from ICP is 1.77%, indicating the successful doping of Pd.

**FIGURE 3:**
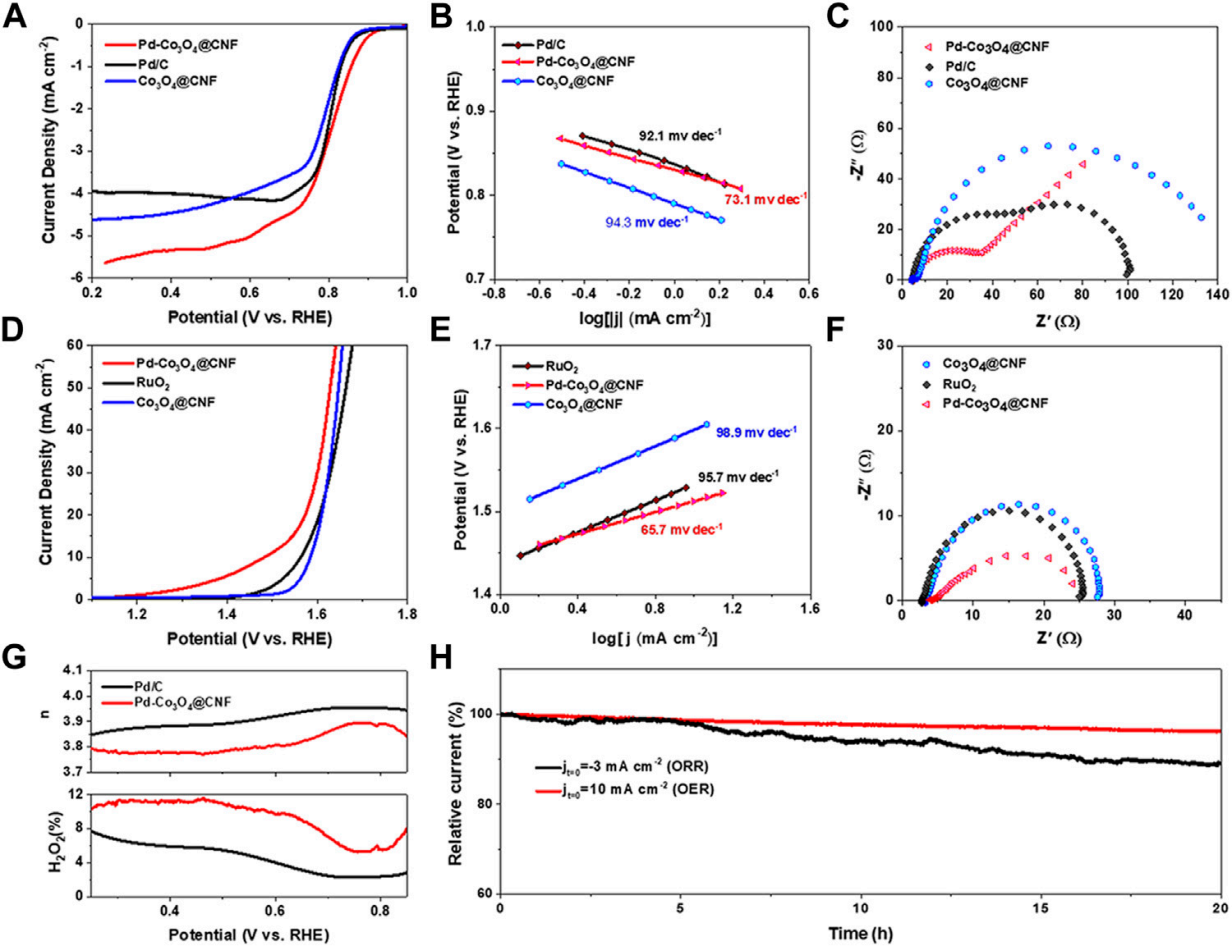
**(A)** Polarization curves, **(B)** Tafel plots, and **(C)** Nyquist plots obtained from EIS measurements for ORR. **(D)** Polarization curves, **(E)** Tafel plots, and **(F)** Nyquist plots obtained from EIS measurements for OER. **(G)** Electron transfer number n (top) and H_2_O_2_ yield (bottom) vs. potential of Pd-Co_3_O_4_@CNF and Pd/C **(H)** CA curves of Pd-Co_3_O_4_@CNF under ORR and OER conditions.

**FIGURE 4:**
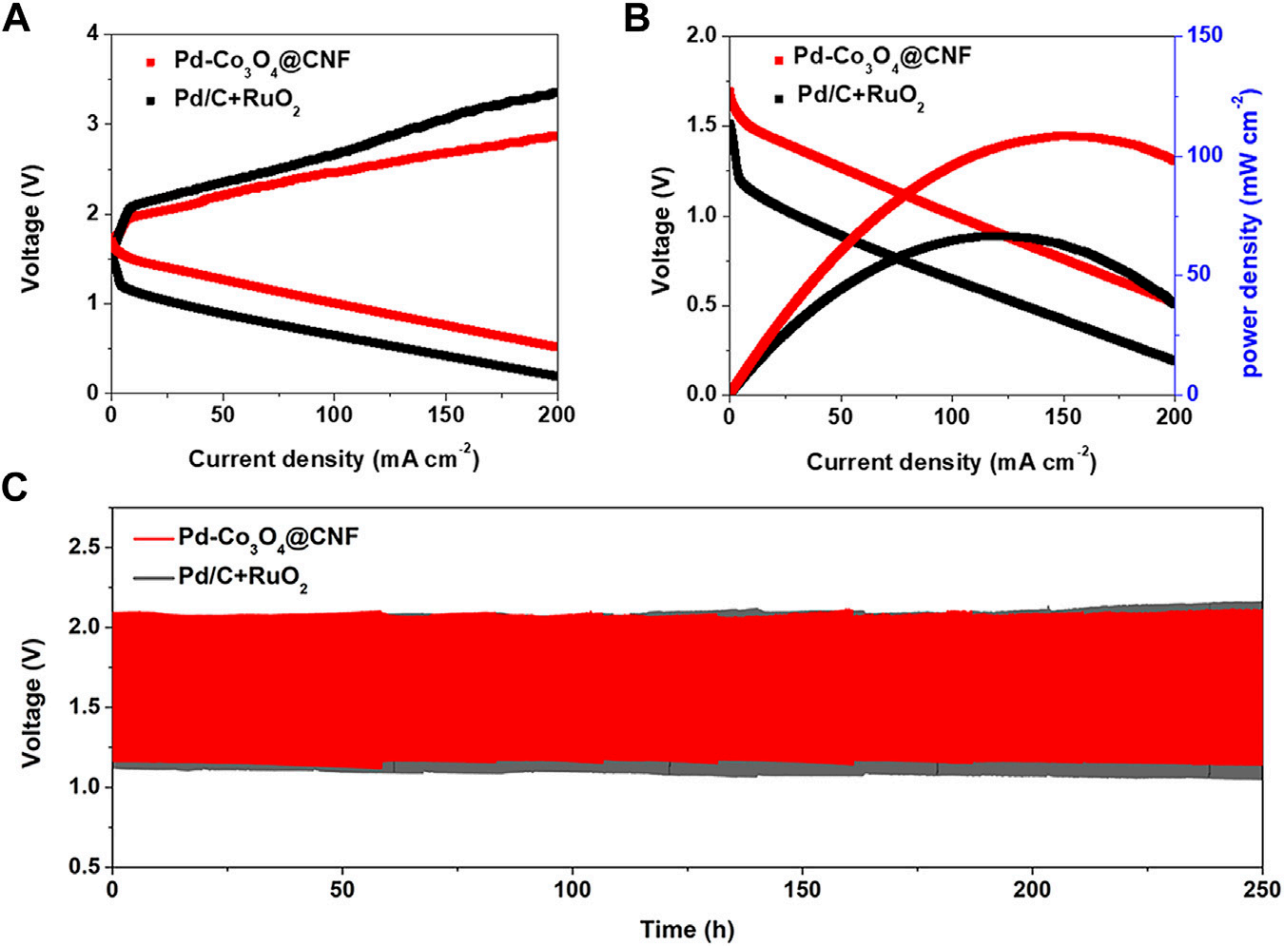
**(A)** Charge and discharge polarization curves. **(B)** Power density and polarization plots. **(C)** Battery cycling test at charging and discharging current densities of 10 mA cm^−2^.

The performance of Pd-Co_3_O_4_@CNF as an oxygen electrode in 1 M KOH was tested by three-electrode system. As shown in Figures 3A,D, LSV polarization curves reflect the ORR and OER activities of catalysts. For ORR, the half-wave potential (E_1/2_) of Pd-Co_3_O_4_@CNF is 0.81 V, which is more positive than Co_3_O_4_@CNF and commercial Pd/C catalyst. For OER, the potential corresponding to current density of 10 mA cm^−2^ (E_j10_) is 1.48 V, compared with 1.56 V for commercial RuO_2_ catalysts. As shown in Figures 4B,E, Pd-Co_3_O_4_@CNF exhibits the lowest Tafel slopes (73.1 mV dec^−1^ and 65.7 mV dec^−1^) for both OER and ORR, revealing the enhanced reaction kinetics (Wang et al., 2021). The Nyquist plots of catalysts as shown in Figures 3C,F demonstrated that Pd-Co_3_O_4_@CNF oxygen electrode has the lowest charge transfer resistance, indicating that the electrocatalysis process is enhanced**.** Moreover, as confirmed in Figure 3G and Supplementary Figure S6, Pd-Co_3_O_4_@CNF shows high 4e^−^ transfer selectivity in ORR process. In addition, the durability of the electrode and stability of its electrochemical performance are also the key criteria for evaluating electrocatalysts. Considering the self-supporting structure and integrated synthesis of the electrode, Pd-Co_3_O_4_@CNF exhibit excellent stability. As shown in Supplementary Figures S7, S8 and Figure 3H, the stability of Pd-Co_3_O_4_@CNF was investigated by ADT and CA tests. The current density of Pd-Co_3_O_4_@CNF oxygen electrode at the same potential did not decrease significantly after 5000 consecutive potential cycles, respectively. In addition, the current density of Pd-Co_3_O_4_@CNF can be maintained over 89% of the initial current density after a 20 h long termed CA test, indicating that Pd-Co_3_O_4_@CNF oxygen electrode can retain most of its activity after a long period of operation, especially compared with commercial catalysts (Supplementary Figure S9).

Under the proposal of oxygen electrode performance discussed above, Pd-Co_3_O_4_@CNF electrode was applied to the air cathode in a rechargeable Zn-air battery as illustrated in Supplementary Figure S10. According to the experimental instructions, air electrodes were also prepared by dropping a mixture of commercial Pd/C and RuO_2_, and the battery performance was tested as a comparison. As shown in Figures 4A,B, the battery based on Pd-Co_3_O_4_@CNF exhibited lower charging voltage, higher discharge voltage, and higher peak power density compared to commercial catalysts, demonstrating the optimized performance of Pd-Co_3_O_4_@CNF electrode. In addition, constant current charge-discharge tests were performed on air cathode at a current density of 10 mA cm^−2^ to appraise the rechargeability of electrodes. As illustrated in Figure 4C and Supplementary Figure S11, Pd-Co_3_O_4_@CNF based ZABs provides a lower charge-discharge gap and maintain over 240 h, and it exhibits superior stability to commercial catalysts-based battery. As mentioned above, the Pd-Co_3_O_4_@CNF air cathode exhibits battery performance in zinc-air cells, which can be attributed to its excellent electrocatalytic activity and stability for both OER and ORR.

## Conclusion

In summary, Pd-Co_3_O_4_@CNF self-supporting electrode was successful prepared by electrospinning method and subsequent thermal treatment. Benefiting from its plentiful active sites, high specific surface area, and integrated configuration, Pd-Co_3_O_4_@CNF exhibits enhanced activity and stability for oxygen electrode reactions in 1.0 M KOH. Stimulated by the promising bifunctional activity, the ZABs with Pd-Co_3_O_4_@CNF electrode exhibit superior charge-discharge capacity and stability compared with the commercial catalysts based ZABs. This study provides an efficient and scalable method of synthesis high-performance self-supporting electrodes by effectively introducing metallic active component. This high performance and stable air cathode has been proven to be effective in ZABs applications. It also proposes a general strategy for manufacturing flexible self-supporting electrodes which can applied to energy storage and conversion devices.

## Data Availability

The original contributions presented in the study are included in the article/Supplementary Material, further inquiries can be directed to the corresponding authors.
