# Supplementary material for: Pd Doped Co3O4 Loaded on Carbon Nanofibers as Highly Efficient Free-Standing Electrocatalyst for Oxygen Reduction and Oxygen Evolution Reactions
Source: Front Chem. 2022 Jan 12;9:812375. doi: 10.3389/fchem.2021.812375 (PMC8789885; doi:10.3389/fchem.2021.812375)
Supplement: Supplementary file 1 [file DataSheet1.docx]

Supplementary Material


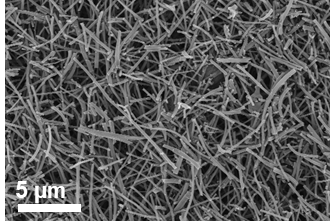


Supplementary Figure S1. SEM images of Pd-Co_3_O_4_@CNF.


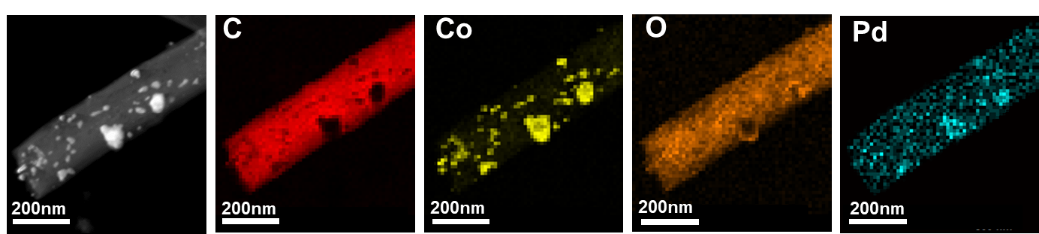


Supplementary Figure S2. HAADF-STEM and the corresponding EDS elemental mapping images of Pd-Co_3_O_4_@CNF.


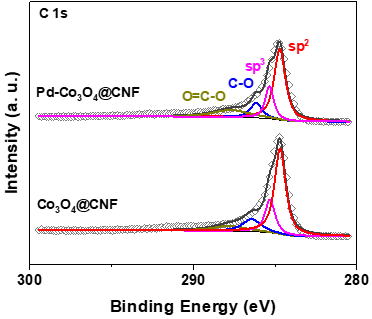


Supplementary Figure S3. C 1s XPS spectra of Co_3_O_4_@CNF and Pd-Co_3_O_4_@CNF.


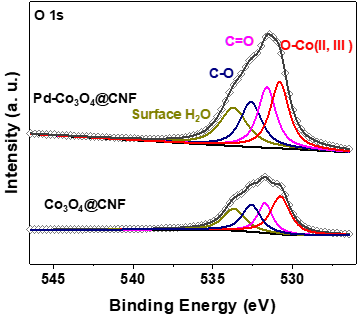


Supplementary Figure S4. O1s XPS spectra of Co_3_O_4_@CNF and Pd-Co_3_O_4_@CNF.


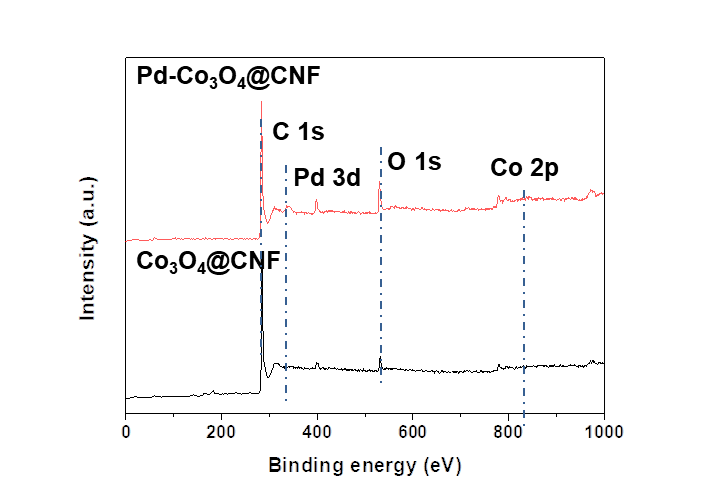


Supplementary Figure S5. XPS survey spectra of Co_3_O_4_@CNF and Pd-Co_3_O_4_@CNF.


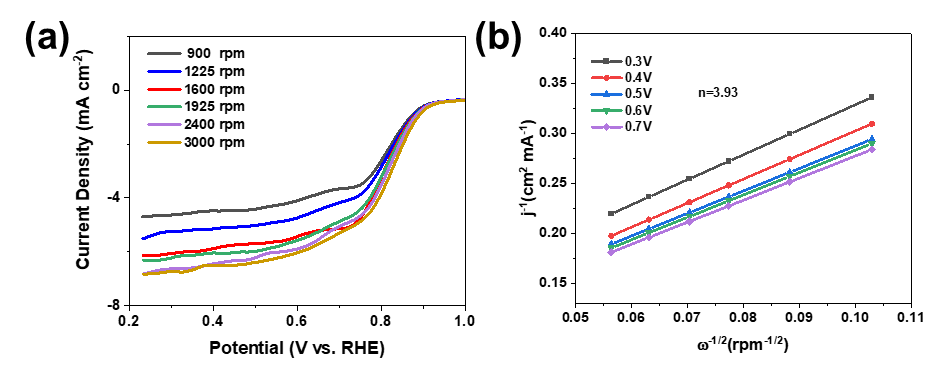


Supplementary Figure S6. (a)RDE polarization curves at various rotation rates and (b) K-L plot of J^-1^ versus ω^-1^ of Pd-Co_3_O_4_@CNF.


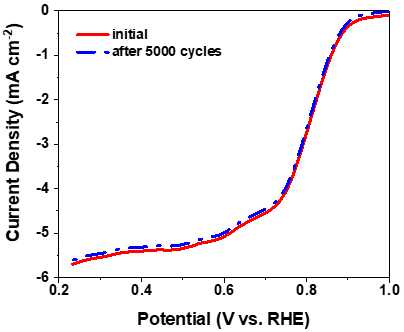


Supplementary Figure S7. LSV curves of Pd-Co_3_O_4_@CNF before and after 50,00 potential cycles under ORR conditions.

Supplementary Figure S8. LSV curves of Pd-Co_3_O_4_@CNF before and after 50,00 potential cycles under OER conditions.

Supplementary Figure S9. CA curves of Pd/C for ORR and RuO_2_ for OER.


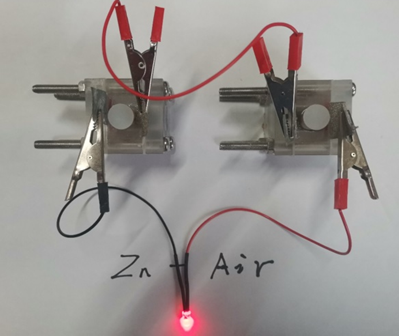


Supplementary Figure S10. Discharge demonstration to power the LED using two primary Zn-air batteries in series using Pd-Co_3_O_4_@CNF as the cathode catalyst.


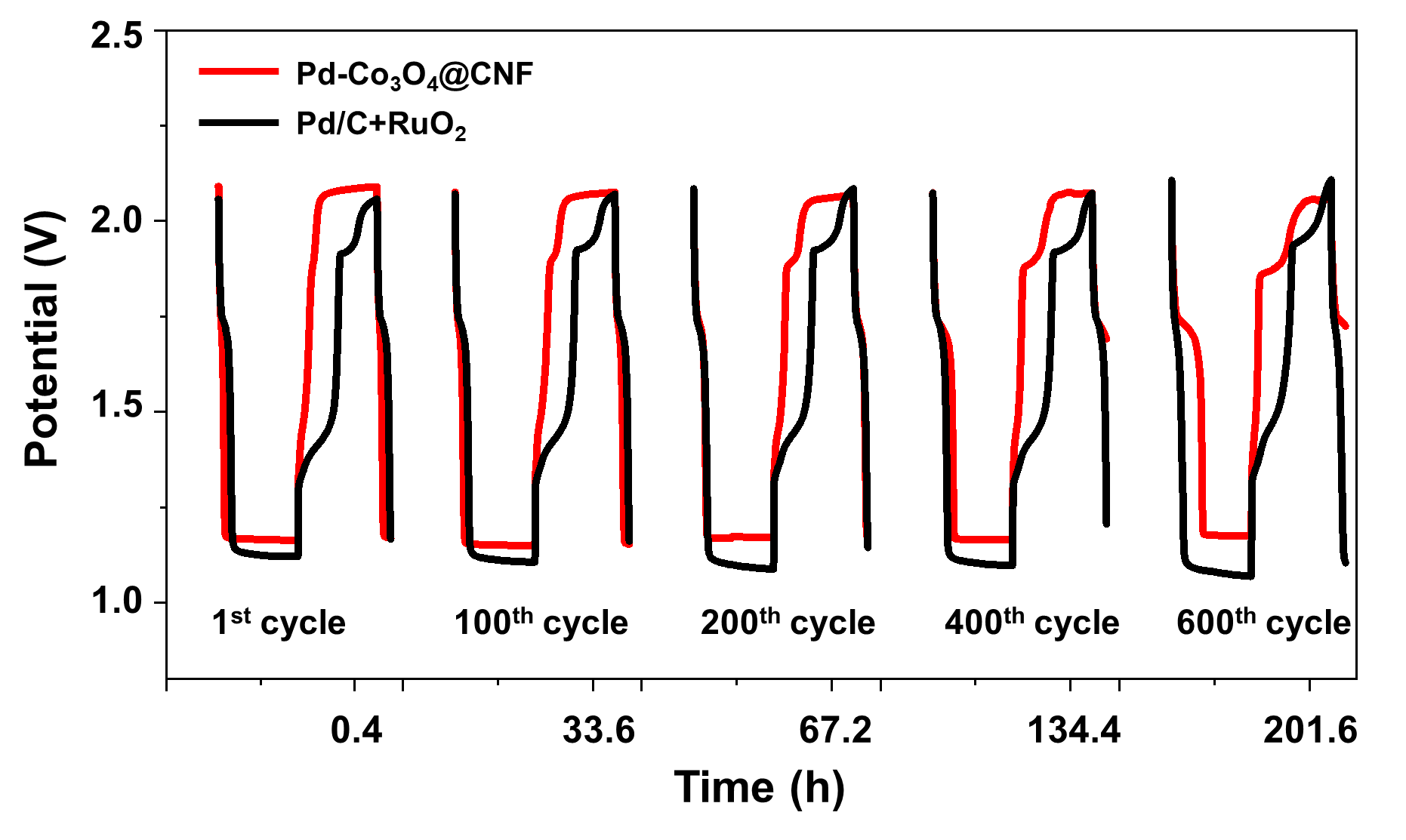


Supplementary Figure S11. 1^st^, 100^th^, 200^th^, 400^th^ and 600^th^ batery cycling test at charging and discharging current densities of 10 mA cm^-2^ (20 min per cycle).

Supplementary Table S1. The surface and bulk elemental composition of Pd-Co_3_O_4_@CNF.

| elemental | Atomic %  (from XPS) | | Weight %  (from XPS) | Atomic %  (from ICP) | Weight %  (from ICP) | |
| --- | --- | --- | --- | --- | --- | --- |
| C | 89.25 | 81.61 | | 90.15 | 81.99 |  |
| O | 9.4 | 11.46 | | 8.23 | 9.98 |  |
| Co | 1.11 | 4.99 | | 1.40 | 6.26 | |
| Pd | 0.24 | 1.94 | | 0.22 | 1.77 | |
